# Acute Liver Injury after CCl_4_ Administration Is Independent of Smad7 Expression in Myeloid Cells

**DOI:** 10.3390/ijms20225528

**Published:** 2019-11-06

**Authors:** Jessica Endig, Ludmilla Unrau, Paulina Sprezyna, Sebasting Rading, Meliha Karsak, Diane Goltz, Lukas C. Heukamp, Gisa Tiegs, Linda Diehl

**Affiliations:** 1 Institute of Experimental Immunology and Hepatology, Center for Experimental medicine, University Medical Center Hamburg-Eppendorf, Martinistrasse 52, 20246 Hamburg, Germany; 2 Neuronal and Cellular Signal Transduction, Center for Molecular neurobiology, University Medical Center Hamburg-Eppendorf, Martinistrasse 52, 20246 Hamburg, Germany; 3 Institute of Pathology, University Hosptital Cologne, Kerpener Str. 62, 50937 Cologne, Germany; 4 Institute for Hematopathology Hamburg, Fangdieckstr. 75a, 22547 Hamburg, Germany

**Keywords:** liver injury, Smad7, TGF-β, myeloid cell, inflammation, regeneration

## Abstract

Myeloid cells are essential for the initiation and termination of innate and adaptive immunity that create homeostasis in the liver. Smad7 is an inhibitor of the transforming growth factor β (TGF-β) signaling pathway, which regulates inflammatory cellular processes. Knockdown of Smad7 in hepatocytes has been shown to promote liver fibrosis, but little is known about the effects of Smad7 in myeloid cells during inflammatory responses in the liver. Using mice with a myeloid-specific knockdown of Smad7 (LysM-Cre Smad7^fl/fl^), we investigated the impact of Smad7 deficiency in myeloid cells on liver inflammation and regeneration using the well-established model of CCl_4_-mediated liver injury. Early (24/48 h) and late (7 d) time points were analyzed. We found that CCl_4_ induces severe liver injury, with elevated serum ALT levels, centrilobular and periportal necrosis, infiltrating myeloid cells and an increase of inflammatory cytokines in the liver. Furthermore, as expected, inflammation peaked at 24 h and subsided after 7 d. However, the knockdown of Smad7 in myeloid cells did not affect any of the investigated parameters in the CCl_4_-treated animals. In summary, our results suggest that the inhibition of TGF-β signaling via Smad7 expression in myeloid cells is dispensable for the induction and control of acute CCl_4_-induced liver injury.

## 1. Introduction

The response of the liver to injury, irrespectively of aetiology, involves different cell types such as hepatocytes, hepatic stellate cells (HSCs) and macrophages (Kupffer cells (KCs)), and encompasses different phases. Characterized by cellular stress leading to various kinds of hepatocyte death, this decline of hepatocytes activates a cascade of signals that initiates inflammation and tissue repair mechanisms. After liver injury, complete repair and regeneration are possible, but if the injury becomes chronic it can also lead to fibrosis, cirrhosis and hepatocellular carcinoma (HCC). 

Hepatocyte damage leading to fibrosis can be induced via oxidative stress that induces activation of HSCs and Kupffer cells in which TGF-β is a key mediator [1]. Signaling via the TβRI leads to the recruitment of receptor-activated Smads (R-Smads), including Smad2 and Smad3, which in turn complex with the common Smad (C-Smad) Smad4. This complex then translocates into the nucleus where it regulates transcription of TGF-β target genes. TGF-β mediated signals can be regulated in several ways. Ubiquitinylation of phosphorylated Smad molecules in the nucleus leads to their proteasomal degradation by which the level of activated Smad proteins is controlled. Furthermore, TGF-β induced target genes include the inhibitory Smad (I-Smads) molecules 6 and 7, of which Smad7 can counteract TGF-β signaling via its association with the TβRI. The interaction of Smad7 with the TβRI can, on the one hand, inhibit the recruitment and activation of the R-Smads. On the other hand, it can lead to TβRI receptor degradation via the recruitment of the ubiquitin E3 ligases Smurf1 and 2 [2]. TGF-β-dependent signals are important regulators of cell proliferation and survival. TGF-β-induced cytostasis functions via the repression of growth-promoting transcription factors (e.g., c-Myc, Id1-3) and the induction of specific CDK inhibitors [3].

Upon liver injury, several hepatic cell types, such as organ-resident macrophages (i.e., Kupffer cells) and hepatic stellate cells (HSCs), readily produce TGF-β. This is pivotal for fibrosis progression, as TGF-β promotes HSC activation and transdifferentiation into myofibroblasts that are then stimulated to proliferate according to further TGF-β signals [1]. Both depletion of hepatic stellate cells [4] and depletion of macrophages [5] results in the attenuation of liver fibrosis, which is characterized by a marked decrease in matrix deposition and a reduction of HSC activation. Moreover, in several liver injury models, interference with pro-fibrotic TGF-β signaling attenuates the severity of disease or injury. Direct inhibition of TGF-β receptor signaling in hepatocytes promotes liver regeneration after acute CCl_4_ intoxication [6]. Expression of the negative regulator of TGF-β signaling Smad7 leads to diminished sensitivity to TGF-β signals. For instance, deletion of Smad7 in hepatocytes aggravates liver injury and steatosis after alcohol feeding [7] as well as fibrogenesis in an acute CCl_4_-mediated injury model [8], and it accelerates chemically induced HCC development [9]. Contrary, hepatic overexpression of Smad7 inhibits fibrogenesis in CCl_4_-mediated injury [10]. 

In liver injury, macrophages produce TGF-β and are important for cross talk with HSCs, leading to the activation and generation of myofibroblasts that produce extracellular matrix components for fibrosis. It is, however, unclear how TGF-β signaling and Smad7-mediated counter regulation of TGF-β signaling is regulated in hepatic macrophages during liver injury and inflammation. Overexpression of Smad7 in intestinal macrophages is associated with their pro-inflammatory activation [11], and shRNA-mediated knockdown of Smad7 is currently being tested as a therapy for inflammatory bowel disease [12]. Here, we investigated the contribution of myeloid-expressed Smad7 on disease severity in an acute model of liver injury and regeneration after a single application of CCl_4_ using a myeloid-specific Smad7 knockdown mouse model (LysM-Cre Smad7^fl/fl^). Our results show that a single application of CCl_4_ in LysM-Cre Smad7^fl/fl^ knockdown and their Smad7^fl/fl^ littermate controls led to liver injury with elevated liver enzymes in the serum, areas of necrosis in histological analyses and infiltration of immune cells, as well as the induction of liver regeneration. However, the knockdown of Smad7 in LysM-expressing cells did not influence the severity of any of these parameters that are indicative of liver injury and regeneration. Thus, negative regulation of TGF-β signaling via Smad7 expression in myeloid cells does not contribute to CCl_4_-mediated liver injury and regeneration.

## 2. Results

### 2.1. Liver Injury after CCl_4_ Administration is Similar in Myeloid-Specific Smad7 Knockdown and Wild-Type Controls

**Figure 1:**
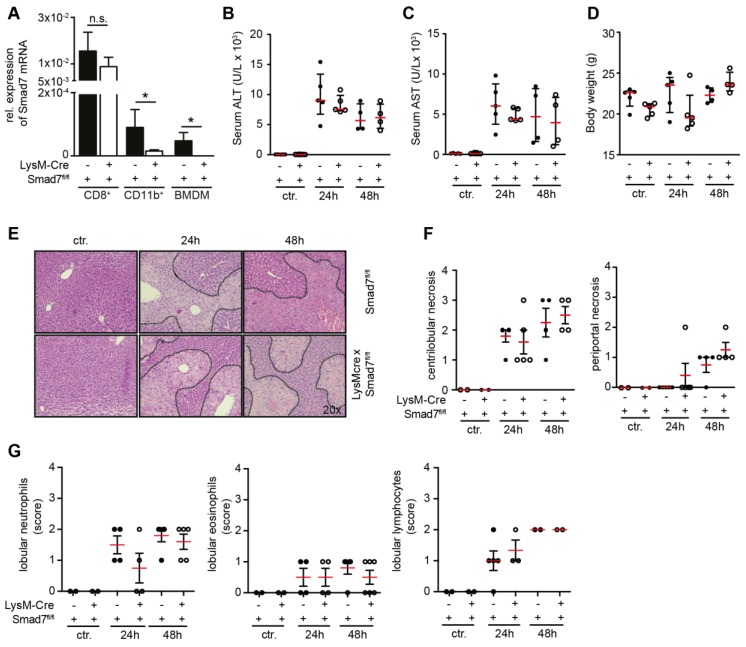
Liver enzymes and histological damage parameters are not changed due to myeloid deletion of Smad7 after CCl_4_ administration. (**A**) Smad7 mRNA expression by quantitative real time PCR on splenic CD11b+, CD8+ and bone-marrow-derived macrophages (BMDMs) from Smad7^fl/fl^ and LysM-Cre Smad7^fl/fl^ littermates (*n* = 4). (**B**–**G**) The 8- to 10-week-old Smad7^fl/fl^ and LysM-Cre Smad7^fl/fl^ littermates were injected with 30% CCl_4_ in corn oil and sacrificed at the indicated time points. (**B)** Serum ALT, (**C**) serum AST, and (**D**) body weight were determined 24 and 48 h after i.p. injection of CCl_4_. (**E**) Representative H & E staining of Smad7^fl/fl^ and LysM-Cre Smad7^fl/fl^ littermates. Representative pictures are shown at 20x magnification. (**F**) Histopathological score of centrilobular and periportal necrosis in H & E sections. (**G**) Histopathological score of lobular neutrophil, lymphocyte and eosinophil infiltration in H & E sections. Data shown are representative of three independent experiments with three to five animals per group. Error bars indicate the mean ± SEM. * *p* ≤ 0.05 calculated by Student’s *t*-test, n.s. = not significant. No other significant *p*-values (≤0.05) between LysM-Cre^pos^ and Cre^neg^ Smad7^fl/fl^ were present in the data from panels (**B**–**D**,**F**,**G**).

TGF-β signaling is pivotal in liver injuries leading to fibrosis [13], and expression of the negative regulator of TGF-β signaling, Smad7, can modulate liver injury, fibrosis and also cancer development in (for instance) hepatocytes [7,9,10]. Hepatic macrophages produce high amounts of TGF-β and are pivotal for fibrosis progression via the activation of HSCs [1]. In order to investigate whether Smad7-dependent modulation of TGF-β signaling influences the severity of liver injury in macrophages, we generated a myeloid-specific Smad7 knockdown mouse model via backcrossing a LysM-Cre mouse line with a floxed Smad7 mouse line, in which the Cre-mediated recombination results in the deletion of exon 1 from the Smad7 gene [14]. As hepatocyte-specific deletion of Smad7 has been described as leading to spontaneous liver dysfunction [7], and TGF-β signaling is in general important for the development of various tissues, we first analyzed whether Smad7 knockdown in LysM-Cre Smad7^fl/fl^ mice was efficient. *Smad7* mRNA was significantly reduced in splenic CD11b^+^ cells and bone-marrow-derived macrophages (BMDMs) from LysM-Cre Smad7^fl/fl^ animals, whereas *Smad7* mRNA was readily detected in both splenic CD11b^+^ cells and BMDMs from Smad7^fl/fl^ control animals and in splenic CD8^+^ T cells from both LysM-Cre Smad7^fl/fl^ and Smad7^fl/fl^ control animals. This indicates that the LysM-Cre-mediated deletion of Smad7 is both highly efficient as well as specific (Figure 1A). 

We injected mice with a single dose of CCl_4_ i.p. and analyzed various parameters at 24 and 48 h after the application. Exposure to CCl_4_ leads to centrilobular hepatic necrosis, due to the generation of highly toxic metabolites via cytochrome P450 2E1 [15]. The subsequent hepatocyte death leads to the release of alanine aminotransferase (ALT) and aspartate aminotransferase (AST). Mice receiving CCl_4_ had markedly increased ALT and AST levels in the serum 24 and 48 h after injection, indicating hepatocyte loss, although their body weight did not change (Figure 1B–D). H & E staining revealed extensive liver injury after CCl_4_ administration (Figure 1E), but detailed analysis could not reveal an impact of Smad7 deficiency on myeloid cells for parameters such as area of necrosis (Figure 1F) or infiltrating immune cells, such as neutrophils, eosinophils or lymphocytes (Figure 1G). In summary, these data indicate that Smad7 expression in myeloid cells does not influence the extent of liver injury nor the composition of infiltrating immune cells after CCl_4_-induced liver damage.

### 2.2. Neither Immune Cell Infiltration nor Inflammatory Gene Expression is Affected by the Loss of Smad7 Expression in Myeloid Cells after CCl_4_ Administration

**Figure 2:**
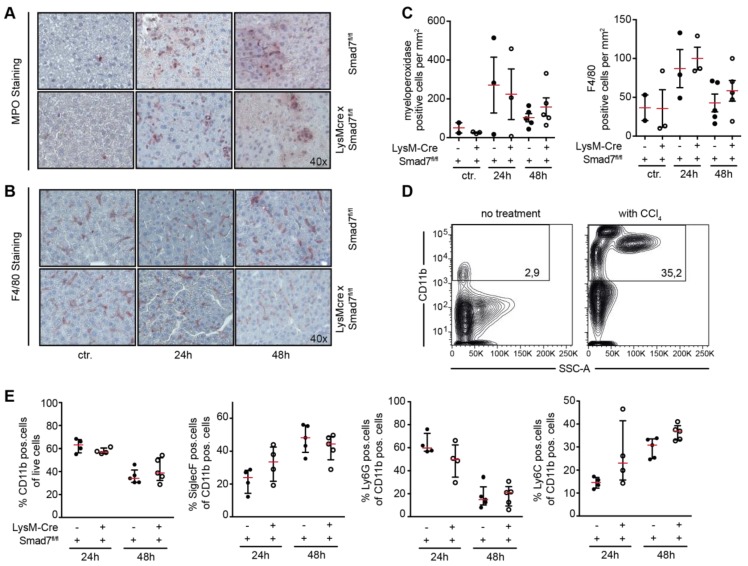
Myeloid cell infiltration after CCl_4_ administration in LysM-Cre Smad7^fl/fl^ mice. The 8- to 10- week-old Smad7^fl/fl^ and LysM-Cre Smad7^fl/fl^ littermates were injected with 30% CCl_4_ in corn oil or left untreated and sacrificed at the indicated time points. (**A**,**B**) Immunohistochemical staining for myeloperoxidase (MPO) and F4/80 in liver sections of mice injected with CCl_4_. Representative pictures are shown at 40x magnification. (**C**) Graphs show enumeration of MPO- and F4/80-positive cells per mm^2^. (**D**,**E**) Non-parenchymal cells (NPC) were isolated and stained for CD11b, CD11c, SiglecF, Ly6G and Ly6C. Staining with a fixable live/dead stain identified living cells. (**D**) Representative dot plots showing percentages (numbers in black rectangles) of viable CD11b^+^ cells in livers from CCl_4_-treated and non-treated mice. (**E**) Percentages of CD11b^+^ cells within live cells and percentages of SiglecF^+^, Ly6G^+^ or Ly6C^+^ cells within CD11b^+^ cells in CCl_4_-treated Smad7^fl/fl^ and LysM-Cre Smad7^fl/fl^ mice after 24 and 48 h. Data shown are representative of three independent experiments with three to five animals per group. Error bars indicate the mean ± SEM. Statistical significance was calculated by ANOVA, but no significant *p*-values (≤0.05) between LysM-Cre^pos^ and Cre^neg^ Smad7^fl/fl^ were present in the data from panels (**C**,**E**).

**Figure 3:**
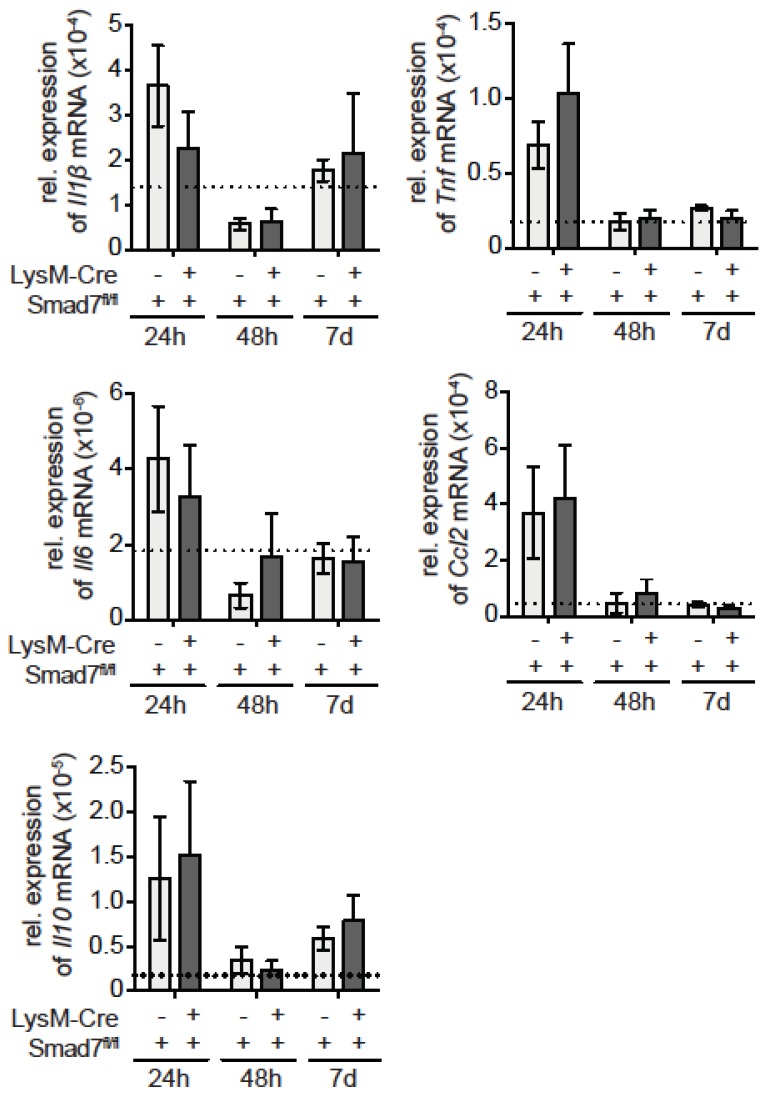
Inflammatory gene expression in LysM-Cre Smad7^fl/fl^ after CCl_4_ injection. The 8- to 10-week-old Smad7^fl/fl^ and LysM-Cre Smad7^fl/fl^ littermates were injected with 30% CCl_4_ in corn oil and sacrificed at the indicated time points. As a control, mice were left untreated. At the indicated times, liver samples were taken for quantitative real-time PCR for pro- (*Il1b*, *Tnf*, *Ifng*, *Il6*, *Ccl2*) and anti-inflammatory (*Il10*) genes. Relative mRNA expression levels of target genes are depicted calculated in relation to the expression of the reference gene mRNA by the ΔCt method. The dotted line indicates expression in non-treated control animals. Data shown are representative of three independent experiments with three to five animals per group. Error bars indicate mean ± SEM. Statistical significance was calculated by ANOVA, but no significant *p*-values (≤0.05) between LysM-Cre^pos^ and Cre^neg^ Smad7^fl/fl^ were present in these data.

We next evaluated in more detail the myeloid cell infiltration into injured livers after CCl_4_ injection. Using histological staining we analyzed the amount of infiltrating myeloperoxidase (MPO)-expressing cells and F4/80-expressing cells into the livers of CCl_4_-treated animals. Both MPO (Figure 2A)- and F4/80 (Figure 2B)-positive cells were increased in the liver after CCl_4_ treatment, but myeloid Smad7 deficiency did not alter their proportion (Figure 2C). To be able to differentiate in more detail between the myeloid cells in the livers of CCl_4_-treated LysM-Cre Smad7^fl/fl^ and Smad7^fl/fl^ animals, we isolated the non-parenchymal cells (NPC) from these livers and stained the cells for CD11b, CD11c, SiglecF, Ly6G and Ly6C. Flow cytometric analyses revealed a large increase in total CD11b-positive cells in CCl_4_-treated animals compared to the non-treated controls (Figure 2D). After 24 h, the majority of myeloid cells were CD11b^+^CD11c^-^SiglecF^-^Ly6G^+^ neutrophils, whereas after 48 h most CD11b^+^ cells were CD11c^-^SiglecF^+^ and CD11c^-^SiglecF^-^Ly6G^-^Ly6C^+^, indicating that in the course of the inflammatory reaction due to CCl_4_ injection, neutrophils, eosinophils and inflammatory monocytes are recruited into the liver (the gating strategy in Figure S1). However, the knockdown of Smad7 in myeloid cells did not affect the recruitment of any of the investigated myeloid subsets (Figure 2E). Although the number of infiltrating myeloid cells may not be affected, their functionality may be affected. In order to elucidate whether loss of Smad in myeloid cells can affect their function, we analyzed inflammatory gene expression in the livers of CCl_4_-treated LysM-Cre Smad7^fl/fl^ and Smad7^fl/fl^ animals via quantitative reverse transcriptase PCR (qPCR) (Figure 3). Expression of mRNA of the proinflammatory genes *Il1b*, *Il6*, *Tnf* and *Ccl2* was highly induced early after CCl_4_. Expression of anti-inflammatory *Il10* mRNA also increased early after CCl_4_ administration. However, we did not observe any effects of Smad7 knockdown in myeloid cells here either. All investigated parameters were similarly regulated in LysM-Cre Smad7^fl/fl^ and Smad7^fl/fl^ animals. Thus, the infiltration of inflammatory myeloid cells and cytokine/chemokine gene expression was not regulated by Smad7 expression in myeloid cells after CCl_4_ application in vivo.

### 2.3. Smad7 Expressing Myeloid Cells Do not Affect the Regenerative Capacity of the Liver after CCl_4_ Mediated Injury

**Figure 4:**
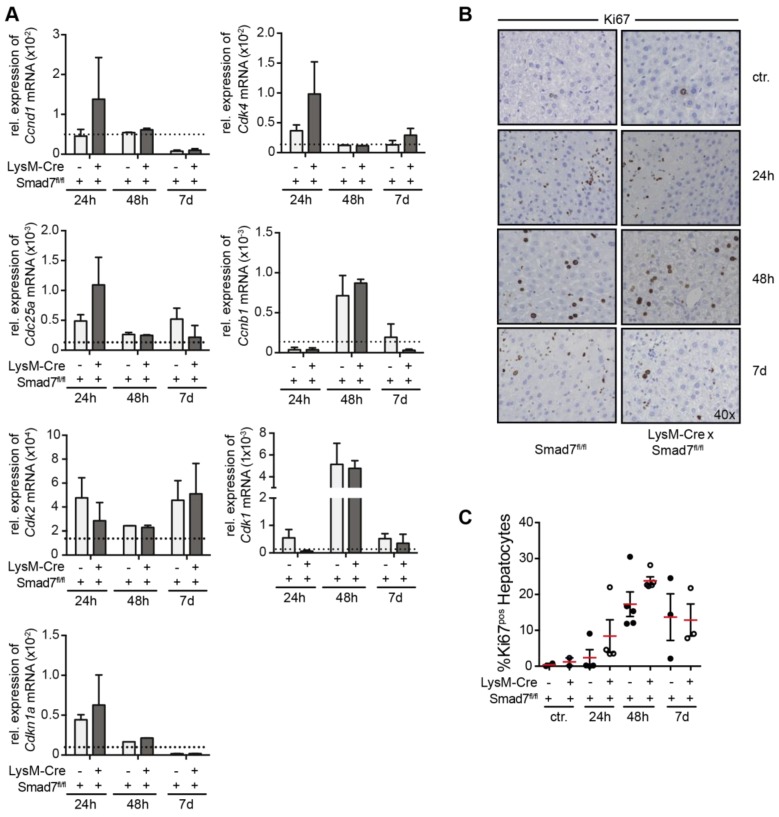
Cell-cycle gene expression in LysM-Cre Smad7^fl/fl^ animals after CCl_4_ administration. The 8- to 10-week-old Smad7^fl/fl^ and LysM-Cre Smad7^fl/fl^ littermates were injected with 30% CCl_4_ in corn oil and sacrificed at the indicated time points. As a control, mice were left untreated. (**A**) At the indicated times, liver samples were taken for quantitative real-time PCR for cell-cycle genes (*Ccnd1*, *Cdk4*, *Cdc25a*, *Cdk2*, *Ccnb1*, *Cdk1* and *Cdk1n1a*). The dotted line indicates expression levels in non-treated control animals. (**B**) Histological staining of the liver section for Ki67. Representative pictures are shown at 200x magnification. (**C**) The percentage of Ki67-positive hepatocytes are shown as % Ki67-positive hepatocyte stained cells per field of view. Data shown are representative of three independent experiments with three to five animals per group. Error bars indicate mean ± SEM. Statistical significance was calculated by ANOVA, but no significant *p*-values (≤0.05) between LysM-Cre^pos^ and Cre^neg^ Smad7^fl/fl^ were present in the data from panels A and C.

Thus far we found no evidence of the involvement of myeloid-expressed Smad7 in the early phases of the immune response after CCl_4_ administration in mice. In addition to the profound and rapid inflammatory response in the liver, administration of CCl_4_ also leads to a well-defined regenerative response [16] that is characterized by the induction of cell-cycle proteins and hepatocyte proliferation during the first seven days after CCl_4_ application. To analyze the regenerative response, we analyzed the expression of several cell-cycle proteins (a.o. cyclins and cyclin-dependent kinases) that are induced to facilitate hepatocyte proliferation and liver regeneration. We could observe early induction of a.o. *Ccnd1*, *Cdk4*, *Cdk2*, *Cdc25a* and *Cdkn1a* mRNA, which was downregulated over the seven-day analysis period. mRNA expression of other cell-cycle proteins such as *Cdk1* and *Ccnb1* reached peak values at 48 h and declined after seven days. However, we could not observe changes in expression levels of these genes due to Smad7 knockdown in LysM-expressing myeloid cells. Corresponding to the mRNA expression analyses of the cell-cycle genes, we found higher levels of Ki67-positive hepatocytes after CCl_4_ injection in histology (Figure 4B), but also here we found no evidence that myeloid-specific Smad7 deficiency affected this response after scoring and counting Ki67 positive hepatocytes (Figure 4C). Together, these data show that Smad7 expression in myeloid cells does not play a role in the liver regenerative response after CCl_4_-induced liver injury.

## 3. Discussion

In the current study, we investigated the impact of Smad7 expression in LysM-expressing myeloid cells in the well-established model of acute liver injury induced by a single injection of the toxin CCl_4_. Smad7 expression is induced by TGF-β-dependent signaling and functions to inhibit further TGF-β signaling. Smad7 can do this via binding to the TβRI, which on the one hand can prevent the binding of further signal transduction molecules, while on the other hand it can mediate the proteasomal degradation of the TβRI [2], both of which lead to the attenuation of TGF-β signaling. The importance of TGF-β signaling in regulating of cells of the immune system is apparent from studies showing that, for instance, deletion of the TβRII in the hematopoietic system leads to the development of a lethal inflammatory syndrome in mice [17]. Furthermore, Smad7 deletion or overexpression in hepatocytes leads to a profound change in susceptibility to CCl_4_- or alcohol-induced liver injury and HCC development [7,9,10]. Although hepatic macrophages produce TGF-β upon activation, and this is thought to promote hepatic stellate cell activation and proliferation leading to fibrosis, the impact of TGF-β signaling within the hepatic myeloid compartment during liver injury is largely unknown. In several inflammatory diseases, like inflammatory bowel disease [18], celiac disease [19] and kidney inflammation [20], high expression of Smad7 is associated with increased disease severity. Smad7 expression leads to the abrogation of TGF-β-mediated immune-suppressive signaling, which normally results in transcriptional suppression of pro-inflammatory cytokine production and inhibition of proliferation. Indeed, Smad7 overexpression in both T cells and macrophages leads to interrupted TGF-β signaling and inflammatory activation, in the gut for instance, and this aggravates disease [11,21]. We evaluated the role of Smad7 expression in myeloid LysM-expressing cells in the context of acute liver injury and inflammation. However, after CCl_4_ injection, all investigated parameters, from liver injury (ALT, AST), necrosis (histology), pro-inflammatory cytokine expression (qPCR) and inflammatory cell recruitment (flow cytometry) into the liver were not altered in myeloid Smad7 knockdown animals, suggesting that in acute CCl_4_-induced liver injury, Smad7-mediated counter regulation of TGF-β signaling in myeloid cells does not play a role. Also, we were unable to find evidence of myeloid-expressed Smad7 involvement in the regenerative response after liver injury. Both myeloid Smad7 knockdown and wild-type control animals had similar levels of hepatic cell-cycle gene expression and comparable hepatocyte proliferation rates. In summary, our data suggest that in acute CCl_4_-mediated liver injury, the absence of Smad7 in myeloid cells does not influence the severity of liver injury or liver regeneration.

## 4. Materials and Methods

### 4.1. Mice

B6.129P2-Lyz2^tm1(cre)Ifo^/J and B6.Cg-Smad7^tm1.1Ink^/J (LysM-Cre Smad7^fl/fl^) mice were backcrossed and bred under specific pathogen-free conditions in the central animal facility of the University Medical Center Hamburg-Eppendorf according to the Federation of Laboratory Animal Science Association guidelines. We used 8- to 12-week-old males for the experiments. The Behörde für Soziales, Familie, Gesundheit und Verbraucherschutz (Hamburg, Germany; approval code 78/16 from 15.09.2016) approved all experiments. All mice were kept under ad libitum supply of food and water and a 12/12 h day–night rhythm. All efforts were made to minimize suffering.

### 4.2. Induction of Acute Hepatitis

CCl_4_ (289116, Sigma Aldrich, Darmstadt, Germany) was injected i.p. at a concentration of 30% CCl_4_ in corn oil (C8265, Sigma Aldrich, Darmstadt, Germany). Mice were sacrificed after the indicated time points. Plasma levels of ALT (20764957322, Roche) and AST (20764949322, Roche, Mannheim, Germany) were measured using a COBAS Integra 400 Plus analyzer from Roche. Liver tissue was isolated for RNA, cDNA synthesis and qPCR to quantify intrahepatic levels of cytokine mRNA or cell-cycle gene mRNA. Histology of liver tissue was performed on formalin-fixed paraffin-embedded liver samples.

### 4.3. Isolation of Non-Parenchymal Liver Cells

Non-parenchymal liver cells were isolated according to standard protocols. In brief, liver was homogenized and washed with phosphate-buffered saline (PBS) after necropsy. Non-parenchymal liver cells were enriched via a 40%/80% Percoll gradient (diluted in PBS, Percoll #17-0891-01, GE Healthcare, Solingen, Germany) gradient (20′ 800×g). The interphase was collected and washed with PBS +2% fetal calf serum (FCS, Sigma, Darmstadt, Germany) and 0.02% sodium azide. Single cells were analyzed by flow cytometry.

### 4.4. Fluorescence-Activated Cell Sorting

Flow cytometric analyses were conducted on a Canto II or LSR II (BD Biosciences, Franklin Lakes, NJ, USA), and data were analyzed using FlowJo software (v10, Tree Star). All antibodies were purchased from Biolegend (San Diego, CA, USA) or eBioscience (ThermoFisher Scientific, Bremen, Germany) unless otherwise stated. A LIVE/DEAD Fixable Violet or Near-IR Dead Cell Stain kit (Invitrogen, ThermoFisher Scientific, Bremen, Germany) was used to exclude dead cells in all samples analyzed. Anti-CD16/32 antibody (clone 93 or 2.4G2) was included in each staining at 10 µ g/mL to block unspecific antibody binding via Fc receptors. All other antibodies were used in a concentration of 1 µg/mL. The LIVE/DEAD Fixable Violet Dead Cell Stain kit (L34955, Invitrogen, ThermoFisher Scientific, Bremen, Germany) was used to exclude dead cells in all samples analyzed. Leukocytes were stained with antibodies against murine CD11b (clone M1/70, PerCP-Cy5.5), CD11c (clone N418, PE-Cy7), SiglecF (clone E50-2440, AlexaFluor647), Ly6G (clone 1A8, FITC) and Ly6C (clone HK1.4, PE).

### 4.5. mRNA Isolation and Quantitative RT-PCR

Murine liver tissue was flash frozen in liquid nitrogen and stored at −80 °C until further processing. mRNA was isolated using a RNeasy Mini kit from Qiagen (#74104, Hilden, Germany) following the manufacturer’s instruction. gDNA was digested separately using the DNase Treatment & Removal kit (AM1906, Invitrogen, ThermoFisher Scientific, Bremen, Germany) from Invitrogen. mRNA was transcribed into cDNA using the High Capacity cDNA Reverse Transcription kit from Applied Biosystems (#4368813). Quantitative RT-PCR (qPCR) was either performed with exon spanning primers for relevant inflammatory and cell-cycle genes using the PowerUp SYBR Green Master Mix (ThermoFisher Scientific, Bremen, Germany) from Applied Biosystems (A25742), or with Taqman gene expression assays (Applied Biosystems, ThermoFisher Scientific, Bremen, Germany) for muSmad7 and the reference genes muGapdh/muHprt1/hu18S on a Viia QuantStudio 7 (ThermoFisher Scientific, Bremen, Germany) (primers used are listed in Table S1). Relative mRNA expression levels were calculated using the ΔCt method.

### 4.6. Histology

Liver tissue was fixed in 4% (*w*/*v*) formalin (#3933, J.T.Baker, Fisher Scientific, Schwerte, Germany) for 24 h at 4 °C, after which it was processed in routine paraffin embedding. Paraffin-embedded liver sections were trimmed (4 µm), deparaffinized in Xylol (#9713.3, Carl Roth, Karlsruhe, Germany) and rehydrated with a descending range of EtOH (100%, 90%, 70% and 50%) and ddH_2_O. Heat-induced antigen retrieval was followed by incubation with Target Retrieval Solution (1x, S1699, Dako, Agilent Technologies, Hamburg, Germany). Cooled slides were washed 1x with Tris-buffered saline (TBS) and blocked with swine serum (S-4000, Vector, 1:10 diluted in Antibody Diluent, S3022, Dako, Agilent Technologies, Hamburg, Germany). Overnight, the specimens were stained with primary antibodies against mouse anti-human Ki67 (1:25, clone B56, BD Biosciences, Franklin Lakes, NJ, USA) rat anti-mouse F4/80 (1:100, MCA497GA, Bio-Rad, Hercules, CA, USA) and goat anti-mouse MPO (1:40, AF3667 R&D systems, Minneapolis, MN, USA) in a humid chamber at 4 °C. Secondary antibody staining was performed with conjugated secondary antibodies against rabbit anti-mouse Ig biotinylated (1:200, #E0354, Dako, Agilent Technologies, Hamburg, Germany), goat anti-rat Ig biotinylated (1:100, BA-9401, Vector Laboratories, Burlingame, CA, USA) and rabbit anti-goat Ig biotinylated (1:200, E0466, Dako, Agilent Technologies, Hamburg, Germany). Bound antibodies were visualized via HRP-Streptavidin (SA-5004, Vector Laboratories, Burlingame, CA, USA) and an AEC staining kit (AEC101-1, Sigma Aldrich, Darmstadt, Germany). Following the color reaction with AEC and washing with ddH_2_O, slides were counter stained with hemalum (1.09249.1000, Merck, Darmstadt, Germany). Heaematoxylin and eosin staining was performed according to Mayer with hemalum and acidified Eosin G (1.15935.0100, Merck, Darmstadt, Germany), followed by an ascending range of EtOH (90%, 96% and 100%) and Xylol incubation prior to mounting.

### 4.7. Statistical Analysis

Student’s *t*- or ANOVA tests were used to determine the statistical significance of the results. Data are depicted as the mean with SEM, and *p*-values < 0.05 were considered significant. 

## Supplementary Materials

Supplementary materials can be found at https://www.mdpi.com/1422-0067/20/22/5528/s1.
