# Supplementary material for: Acute Liver Injury after CCl4 Administration Is Independent of Smad7 Expression in Myeloid Cells
Source: Int J Mol Sci. 2019 Nov 6;20(22):5528. doi: 10.3390/ijms20225528 (PMC6888233; doi:10.3390/ijms20225528)
Supplement: Supplementary file 1 [file ijms-20-05528-s001.pdf]

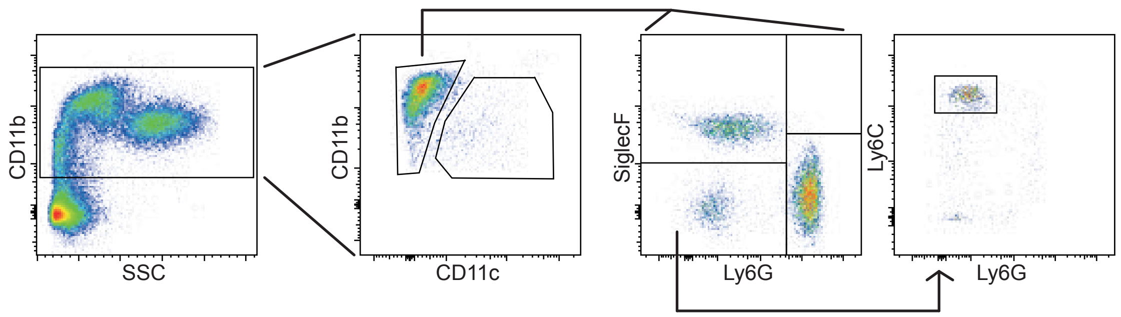


Supplementary Figure S1: Gating strategy for myeloid cell populations in the liver.

**Supplementary Table S1:** Primers used for quantitative reverse transcriptase PCR

| | |
|---|---|
| <u>Cytokine genes</u> | |
| muIl1b forward: | 5'-TGCCACCTTTTGACAGTGATGA-3' |
| muIl1b reverse: | 5'-TGATGTGCTGCTGCGAGATT-3' |
| | |
| muIl6 forward: | 5'-AAAGACAAAGCCAGAGTCCTTCA-3' |
| muIl6 reverse: | 5'-GGAGAGCATTGGAAATTGGGGTA-3' |
| | |
| muTnf forward: | 5'-CCACCACGCTCTTCTGTCTAC-3' |
| muTnf reverse: | 5'-AGGGAGGCCATTTGGGAACT-3' |
| | |
| muCcl2 forward: | 5'-TGGAGCATCCACGTGTTGG-3' |
| muCcl2 reverse: | 5'-ACCTCTCTCTTGAGCTTGGTG-3' |
| | |
| muIl10 forward: | 5'-ATAAGAGCAAGGCAGTGGAGC-3' |
| muIl10 reverse: | 5'-AAGATGTCAAATTCATTCATGGCCT-3' |
| | |
| <u>Cell cycle genes</u> | |
| muCcnd1 forward: | 5'-TCAAGTGTGACCCGGACTG-3' |
| muCcnd1 reverse: | 5'-CAGCCTCTTCCTCCACTTCC-3' |
| | |
| muCdk4 forward: | 5'-GAGCGTAAGATCCCCTGCTT-3' |
| muCdk4 reverse: | 5'-ACCGACACCAATTTCAGCCA-3' |
| | |
| muCdk2 forward: | 5'-CATCTTTGCTGAAATGCACCTAGT-3' |
| muCdk2 reverse: | 5'-TCCTTGTGATGCAGCCACTT-3' |
| | |
| muCdc25a forward: | 5'-GCGGTGTTGAAGAGAGCAGA-3' |
| muCdc25a reverse: | 5'-GAGACTGGGATGGAAGCTGG-3' |
| | |
| muCdkn1a forward: | 5'-AGTGTGCCGTTGTCTCTTCG-3' |
| muCdkn1a reverse: | 5'-AAGTTCCACCGTTCTCGGG-3' |
| | |
| muCdk1 forward: | 5'-CACACGAGGTAGTGACGCTG-3' |
| muCdk1 reverse: | 5'-TCTGAGTCGCCGTGGAAAAG-3' |
| | |
| muCcnb1 forward: | 5'-CAACGGTGAATGGACACCAA-3' |
| muCcnb1 reverse: | 5'-TATGTACAGGCGGCACATGG-3' |
| | |
| <u>Reference genes</u> | |
| mu18S forward: | 5'-CACGGCCGGTACAGTGAAAC-3' |
| mu18S reverse: | 5'-AGAGGAGCGAGCGACCAA A-3' |
| | |
| muHprt1 forward: | 5'-TGCTGACCTGCTGGATTACATT-3' |
| muHprt1 reverse: | 5'-CTTTTATGTCCCCCGTTGACTG-3' |
| | |
| TaqMan gene expression assays: | |
| muSmad7: | Mm00484742_m1 |
| muGapdh: | Mm99999915_g1 |
| hu18S: | Hu03003631-g1 /Hs99999901_s1 |
